# Supplementary material for: Identification of photoperiod-induced specific miRNAs in the adrenal glands of Sunite sheep (Ovis aries)
Source: Front Vet Sci. 2022 Jul 22;9:888207. doi: 10.3389/fvets.2022.888207 (PMC9354845; doi:10.3389/fvets.2022.888207)
Supplement: Supplementary file 1 [file Data_Sheet_1.ZIP › Supplementary file/miRDeep2 file.zip!/miRDeep2 file/Novel_Structure/SP42A1/Novel_704.pdf]

Provisional ID	:	NC_019484.2_26375
Score total	:	15.8
Score for star read(s)	:	3.9
Score for read counts	:	8.3
Score for mfe	:	2
Score for randfold	:	1.6
Score for cons. seed	:	
Total read count	:	28
Mature read count	:	22
Loop read count	:	0
Star read count	:	6

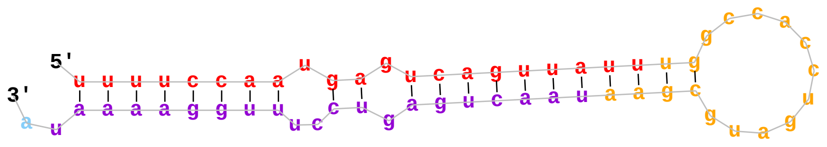

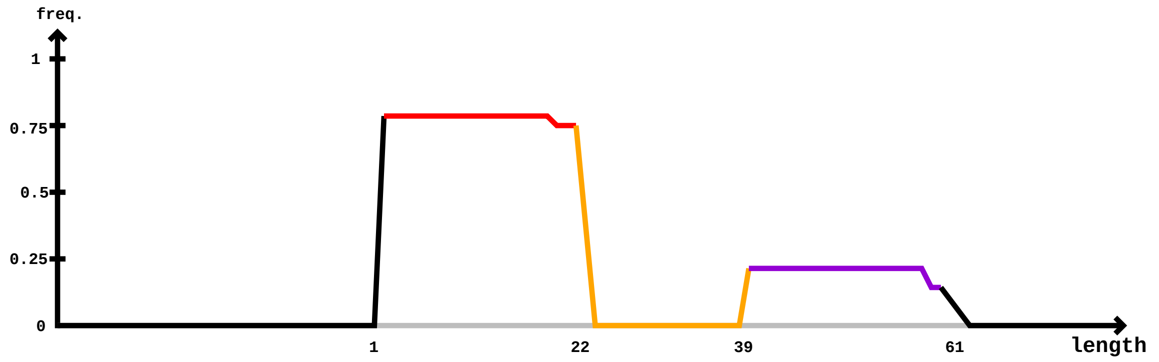

Mature **Star**

```
5'- ucucccuguuuuaaaacuucaacaucaggguc uuuuccaaugagucaguuauu uggccaccugaugcgaa uaacugaguccuuuggaaaau acccugaugcugggaaag   -3'    obs
```

```
5'- ucucccuguuuuaaaacuucaacaucagggucuuuuccaaugagucaguuauuuggccaccugaugcgaauaacugaguccuuuggaaaauacccugaugcugggaaag   -3'    obs
    ucucccuguuuuaaaacuucaacaucagggucuuuuccaaugagucaguuauuuggccaccugaugcgaauaacugaguccuuuggaaaauacccugaugcugggaaag          exp
    ..((((.(((....))).....(((((((((.((((((((.((.(((((((((((...........))))))))))).))..)))))))).)))))))))..))))...    reads    mm    sample
    ................................uuuuccaaugagucaguu...........................................................    1        0     seq
    ................................uuuuccaaugagucaguuaCu........................................................    1        1     seq
    ................................uuuuccaaugagucaguuaGu........................................................    2        1     seq
    ................................uuuuccaaugagucaguuauu........................................................    14       0     seq
    ................................uuuuccaaugagucaguuauuu.......................................................    1        0     seq
    ................................uuuuccaaugagucaguuauuA.......................................................    3        1     seq
    ......................................................................uaacugaguccuuuggaaa....................    2        0     seq
    ......................................................................uaacugaguccuuuggaaaau..................    3        0     seq
    ......................................................................uaacugaguccuuuggaaaaua.................    1        0     seq
```
